# Controversies in the Treatment of Ingrown Nails

**DOI:** 10.1155/2012/783924

**Published:** 2012-05-20

**Authors:** Eckart Haneke

**Affiliations:** ^1^Department of Dermatology, Inselspital, University of Bern, Freiburgstrasse 14, 3010 Bern, Switzerland; ^2^Dermatology Practice Dermaticum, Freiburg, Germany; ^3^Centro de Dermatología Epidermis, Instituto CUF, Porto, Portugal; ^4^Department of Dermatology, Acad Hospital, University of Ghent, Gent, Belgium

## Abstract

Ingrown toenails are one of the most frequent nail disorders of young persons. They may negatively influence daily activities, cause discomfort and pain. Since more than 1000 years, many different treatments have been proposed. Today, conservative and surgical methods are available, which, when carried out with expertise, are able to cure the disease. Packing, taping, gutter treatment, and nail braces are options for relatively mild cases whereas surgery is exclusively done by physicians. Phenolisation of the lateral matrix horn is now the safest, simplest, and most commonly performed method with the lowest recurrence rate. Wedge excisions can no longer be recommended

## 1. Introduction

Ingrown toenails are a common condition of school children and young adults but may be observed at virtually any age. Their treatment is often frustrating for the patient as it may be associated with considerable and long-lasting morbidity and quite frequently with permanently distorted toes and nails.

## 2. Terminology

The controversy begins already with the term: whereas most physicians call the condition ingrown or ingrowing nail (unguis incarnatus) since the nail plate is believed to be the cause [1], others insist that it should be named onychocryptosis as the nail is only covered by hypertrophic lateral nail wall tissue [2]. 

## 3. Types and Aetiopathogenesis of Ingrown Nails

**Figure 1:**
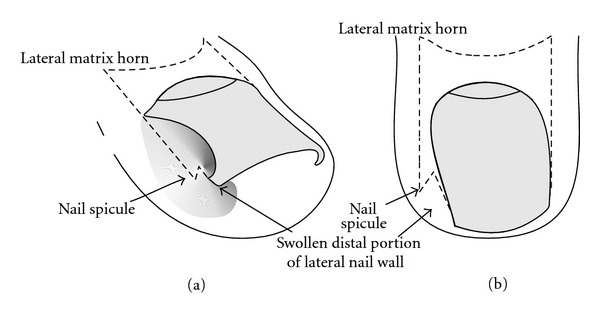
Schematic illustration of the adolescent type of ingrown nail. (a) Oblique view. (b) Dorsal view.

There are several different types of ingrowing nails (Table 1). The most common form is distal-lateral ingrowing. The aetiopathogenesis is usually a wide, relatively markedly curved nail plate, the distal lateral corners of which have been cut obliquely leaving a tiny spicule that digs into the lateral nail groove and finally pierces the epidermis when the nail grows forward (Figure 1). This causes a foreign body reaction with inflammation, granulation tissue, secondary bacterial colonization, and eventually infection [2]. Precipitating factors are narrow pointed shoes, tight socks, hyperhidrosis, juvenile diabetes mellitus, and many more [1].

**Figure 2:**
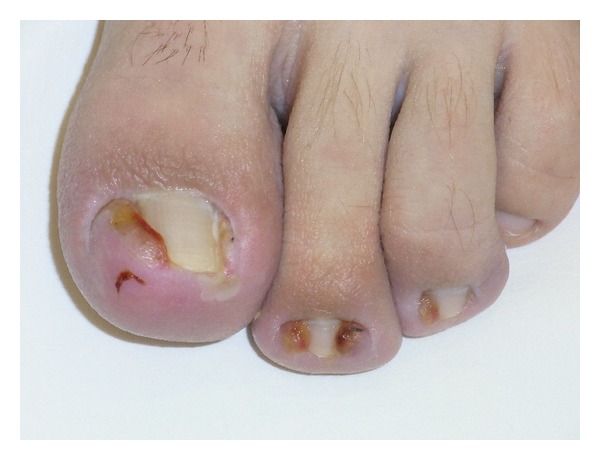
Laterally ingrown nail with granulation tissue in a 15-year-old male patient.

In the most common form, ingrowing usually starts at the distal end of one or both of the lateral nail grooves. The tip of the toe is compressed in a narrow tipped shoe, and when the nail is cut short or the distal corner has been cut off, the distal nail bed is allowed to shrink so that there is no more enough space for the regrowing wide nail (Figure 2). It pushes on the soft tissue which may first react with a circumscribed, usually painful hyperkeratosis called onychophosis. The patient tries to relieve the discomfort by cutting more of the nail corner; however, in order to cut the nail smoothly, one would have to insert the tip of the scissors far deeper, which in turn would mean that one would have to pierce oneself into the soft tissue. This is painful and not done, thus a hook-like piece of the lateral border is left. When the nail grows out, the hook pierces into the nail groove causing even more pain. A vicious cycle of pain, attempt to relieve it, wrong nail cutting, and aggravating the condition is initiated. 

In the elderly, the nail, most often of the hallux, is often sharply bent at its lateral and/or medial margin(s) thus pressing on the nail groove. Again, an onychophosis may be the result, but the nail may also break the integrity of the nail groove epidermis with resultant inflammation, which is usually less marked than in the juvenile type of ingrown nails.

**Figure 3:**
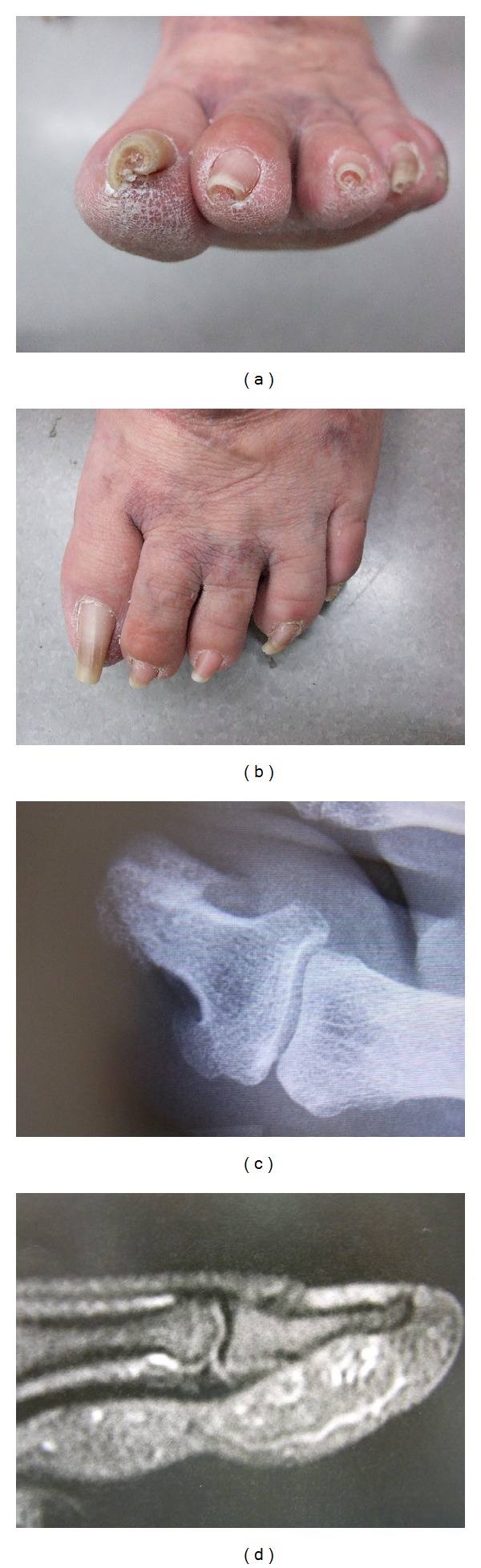
Pincer nails in a 56-year-old female patient. (a) Frontal view. (b) Dorsal view. (c) X-ray dorsal view of the distal phalanges shows the lateral deviation of the terminal phalanges and the medial hook-like exostoses at the base of the bone. (d) X-ray lateral view demonstrates the distal dorsal traction osteophyte.

From adulthood on, many people develop a progressive transverse overcurvature that pinches the nail bed—hence the term pincer nail or unguis constringens—and heaps its distal part up (Figure 3). Often, it remains painless even though the nail may form a complete tube, but sometimes patients describe excruciating pain requiring treatment. The symmetrical form of pincer nails is probably a complex dominant genetic trait with the phalangeal bones being at fault for the development of the overcurvature [3]. Systematic X-ray investigations have shown that there is always a very wide base of the distal phalanx with osteophytes that are bigger on the medial than on the lateral aspect. Usually the whole distal phalanx of the hallux shows a lateral deviation whereas the involved lesser toes point medially. The nail matrix is intimately attached to the base of the terminal phalanx, and with its widening it becomes uncurved proximally which automatically causes overcurving distally. The heaped-up distal portion of the nail bed pulls the soft tissue up resulting in a traction osteophyte [4].

**Figure 4:**
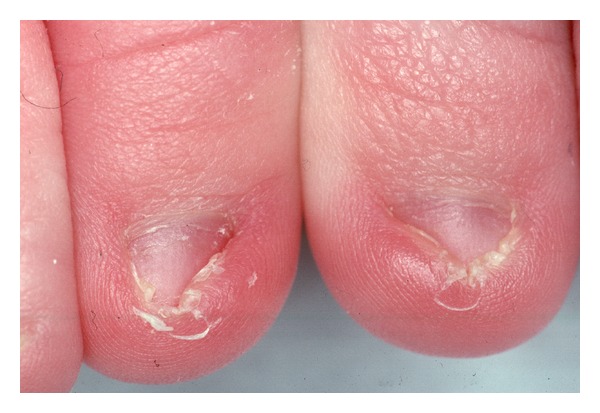
Neonatal ingrown nails.

 In neonates, the hallux nail may not yet have overgrown the distal rim completely which then may grow in either distally or distal laterally (Figure 4). 

Infants sometimes present with a grossly hypertrophic medial nail wall that covers up to one half of the nail. Keratotic debris is kept in the deep crypt between the nail and nail fold and when the debris is degraded by bacteria and yeasts an inflammation develops.

A relatively common condition is congenital malalignment of the big toenail. From birth or shortly thereafter, the nail appears discoloured, thickened, triangular, and oyster-shell like. Probing reveals considerable onycholysis, the degree of which is probably the most important prognostic factor [5]. About one half of the cases is said to show spontaneous resolution; however, in those without improvement and treatment, the condition will result in early onychogryphosis. Roentgenographic investigations have shown structures that were interpreted as a hypertrophic dorsal extension of the lateral ligament of the distal interphalangeal joint ending in the lateral matrix. This was thought to exert a constant pull on the lateral matrix horn with a resultant lateral deviation of the big toenail [6].

## 4. Most Common Types of Ingrown Nails

In this manuscript, the most common form, the distal lateral ingrowing, will be discussed.

In the adolescent type, three stages of ingrown nail are differentiated [7–9]:

1. stage one: inflammation, swelling, and pain,
2. stage two: inflammation, pain, nonhealing wound and oozing, and granulation tissue,
3. stage three: plus abscess formation and chronic induration of the lateral nail fold.

There is often a fluctuation between stages one to three depending on the patient's care of his ingrown toenail. Treatment depends widely on the degree of inflammation.

## 5. Treatment of Ingrown Toenails

The controversy begins with the acknowledgment of a particular aetiology and whether it should be conservative or surgical. Naturally, the latter depends on the specialty of the treating person; podologists and pedicurists favour braces and similar devices, and some dermatologists use other non-invasive approaches whereas the majority tends to perform surgery.

### 5.1. Conservative Approach

Physicians favouring noninvasive treatments consider the aetiopathogenesis of ingrowing nails to be due to a condition amenable to protecting the lateral nail fold from the offending distal nail edge. There are several different methods to achieve this goal, all of which require excellent patient compliance.

#### 5.1.1. Taping

**Figure 5:**
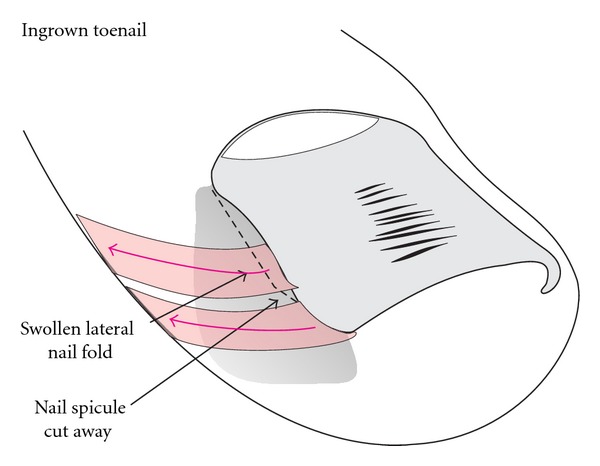
Schematic illustration of taping.

Taping is the least aggressive method. It uses tape to pull the lateral nail fold away from the offending lateral nail edge. Performed correctly and consistently, it can indeed achieve its goal in mild cases of ingrown nails [10]. The technique of taping is, however, crucial, and most patients require repeated education how to perform it. An elastic strip of tape, approximately 15 to 20 mm wide and 5 cm long, is cut and applied so that it allows the lateral nail fold to be pulled away from the nail (Figure 5). This is usually done in an oblique and proximal direction over the the pulp of the toe without impairing the joint movement and avoiding a circular constriction of the toe. A second, so-called anchor tape is applied over the beginning of the first one to fix it and exert even more pull on the distal nail fold [11]. The problem is with toenails that have caused granulation tissue as this is wet, and the tape does not remain stuck on this area. Wiping and drying it with acetone may be of help but is often not enough. Several tape layers may absorb some humidity [12]. In summer time, sweating will also impair the sticking of the tape. Here, we recommend to use a very thin layer of mastisol, which makes the skin more sticky.

#### 5.1.2. Packing

**Figure 6:**
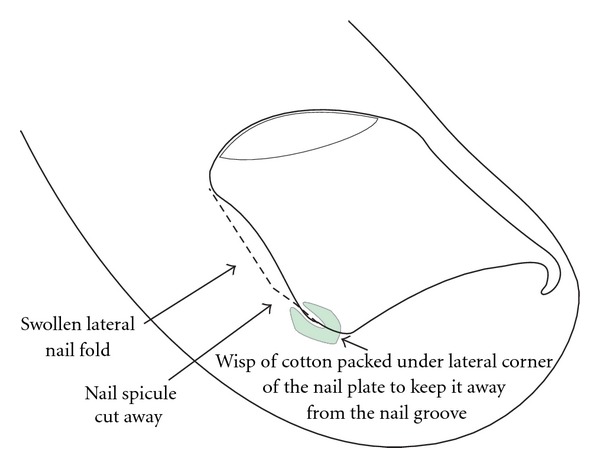
Schematic illustration of packing.

Packing is another simple method. A wisp of cotton is inserted between the corner of the nail and the nail fold (Figure 6). This may first be a bit painful but the patients usually report immediate relief as soon as it has been done. The cotton may be soaked with an antiseptic or disinfectant. The procedure is repeated on a daily basis, each time trying to use a bit more cotton. When complete painlessness is achieved and the nail margin is no longer digging in, the cotton may be fixed with acrylic glue and stay on for a week or so. The treatment period is long, but the results in stage 1 patients are good [13]. Consistent good care is necessary to avoid recurrences. 

#### 5.1.3. Dental Floss

Instead of cotton, dental floss was inserted under the nail corner in order to separate it from the nail groove [14].

#### 5.1.4. Gutter Treatment

**Figure 7:**
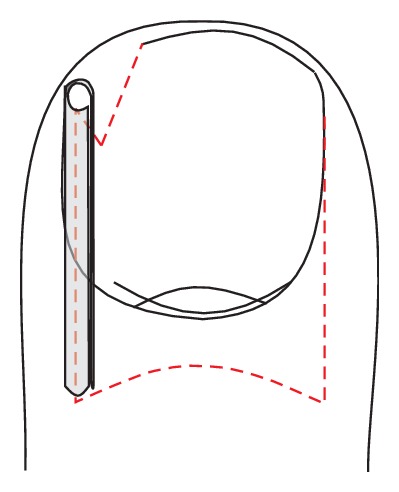
Schematic illustration of gutter treatment.

Gutter treatment is the insertion of a small guard between the lateral nail margin and the nail fold [15, 16]. A sterile plastic tube, most commonly from an intravenous drip infusion, is cut lengthwise to open it. Under local anaesthesia, the nail corner is elevated and the lateral nail margin freed so as to allow the gutter to be slid over it (Figure 7). In contrast to the original publication [15], we do not excise the granulation tissue. The gutter is fixed with one or two stitches [15], tape or acrylic glue [16]. It is important not to cut the spicule of the nail as it gives additional support to the gutter (Figure 7) [16]. This is left in place for a period of 6 to 8 weeks or even longer, during which the inflammatory changes will have subsided. The gutter not only protects the lateral nail groove, but also exerts some pressure on it making the granulation tissue disappear even faster [16].

#### 5.1.5. Nail Braces and Similar Devices

Nail braces are designed to open the curvature of the nail. Their main field of indication is nail overcurvature leading to pincer nail. However, as flat nails rarely grow in the marked curvature apparently plays an aetiological role and is the main treatment goal of many podologists. The braces are made from steel wire or plastic bands. The wire is applied over the dorsal surface of the nail and hooked under its lateral edges. By tightening it, for example, by screwing, the curvature is decreased [17]. Plastic bands are glued on the nail and due to their memory will gently uncurve the nail [18]. Copper-aluminum-manganese-based shape memory alloys have a similar effect [19, 20].

Superelastic wire also uncurves the nails.

Nail ironing is a technique that uses hot haemostat clamps to unbend the nails.

#### 5.1.6. Antibiotics

Many physicians give antibiotics when a patient presents with inflammation and granulation tissue. In my view, this is almost always a useless waste of resources as the nail that digs into the soft tissue is the cause of both the inflammation and granulation tissue. No nail has ever been shown to be sensitive even to the most powerful antibiotic.

Many surgeons who still use cold-steel surgery, particularly wedge excisions, routinely administer antibiotics. This may be justified as they cut through a heavily contaminated area deep into the periungual tissue.

#### 5.1.7. Hygienic Measures

Foot baths and consistent foot hygiene are important factors during conservative treatment, to maintain its effect and as a preparation for surgery. Virtually all ingrown nails present with inflammation and consecutive bacterial colonization, the latter being considerably reduced by disinfective baths and removal of putrid scabs.

### 5.2. Surgical Treatments

The number of surgical methods for the treatment of ingrown nails is huge; probably, there is hardly anyone knowing them all. New or presumedly new methods continue to be published. Many of them are just minor variations of old surgical techniques and very frequently do not bring the slightest progress. Often, they show that the authors do not understand the aetiology and pathogenesis of this condition.

There are two fundamentally different approaches.

- Those authors believing that the soft tissue is primarily at fault propose to take away the soft tissue so that there remains no substrate for the nail to grow in [21–23].
- Most authors favour the view that a wide nail in relation to a narrow nail bed, whatever the cause, is the primary event and consequently propose narrowing of the nail plate so that it does no longer grow in [24].

Elevation of the lateral nail margin and excision and cautery of the granulation tissue of the nail fold were already described by Paul Aegineta (625–690) and Abu al-Qasim, also known as Abulcasis (936–1013). Ambroise Paré (1510–1590) surgically excised it. Fabrizius ab Aquapendente (1537–1619) excised and avulsed the ingrowing nail margin. Almost 180 years ago, the chiropodist Lewis Durlaker (1792–1864) reviewed the “almost savage practices generally employed in the cure of the affections of the nails, which although of a most painful and harassing nature and which frequently lead to distressing and serious results, have been too frequently considered as holding a very humble rank in the catalogue of disease” [25]. Michaelis gave a detailed description of various treatment methods as early as 1830, [26] on which Emmert later based his surgical treatment [27]. Gosselin in 1853 had already counted 75 different varieties of local treatment and described a method, by which an elliptical wedge-shaped piece of nail matrix and skin including the whole nail groove along the edge was removed [28, 29]. The Bernese surgeon Emmert in 1869 and 1884 proposed a wedge excision of the lateral nail wall, groove, adjacent nail, and matrix [30], which is in fact the method proposed by Baudens in 1850 [31]. This is still the intervention most commonly performed by surgeons for the treatment of ingrown nails, particularly in Germany and Switzerland; here it is called Kocher's operation although Kocher had explicitly warned against this method. It was also Emmert who had first described the three stages of ingrown toenails [27]. In the late 1800s, there were more similarly radical surgical operations such as those of Hildebrandt 1884 [32]. Anger's method was to cut a section of the toe from its extremity back to beyond the matrix with cuts extending to the bone [33]. Foote wrote in 1899 that “this operation is a pretty serious one … No one would ever think of removing with an ingrown wisdom tooth, the overlying portion of the cheek. Yet that is exactly done to the toe in all reported methods” [29]. He also noted that there are three ways to remove the cause, whether the nail grows down into the flesh or the flesh grows up against the nail; something may be interposed between the nail and the flesh—what is now known as packing—; the nail may be removed from the flesh; the flesh may be removed from the nail. He proposed an incision to be carried out through the nail beginning at its free end and running parallel to the ingrowing edge, through the skin and matrix to allow skin flaps overlying the matrix to be reflected. The matrix attached to the nail strip was dissected [29]. This is in fact the first description of a selective matrix horn resection. In 1887, Quénu performed a radical nail bed and matrix ablation [34], a method that became later known as Zadik's procedure [35]. The terminal Syme operation is even more radical and is in fact an amputation of the tip of the toe [36, 37].

This short historical overview demonstrates what ingrown toenail sufferers had to face in the past. It is a shame that many of these obsolete methods are still performed by surgeons and other physicians treating ingrown nails although the most reasonable technique had already been described [29]. In the following, some methods will be briefly discussed; it is not possible to deal with all of those ever described.

#### 5.2.1. Nail Avulsion

Nail avulsion causes significant postoperative morbidity. When the nail regrows, the plate is still as wide as it was before and will therefore grow in again. Further, during the period where there was no plate, the nail bed usually shrinks both longitudinally as well as transversely. Absence of the big toenail leads to dorsal dislocation of the most distal portion of the pulp of the toe with a resultant false distal nail wall because of lack of counterpressure of the nail plate during gait. For a fraction of a second, the entire body weight is on the tip of the big toe plus the kinetic energy of the forward thrust resulting in two to two-and-a-half-fold the body weight. This is even more during sports activities. Once there is a distal nail wall, the nail plate cannot overgrow it. The matrix continues to produce nail substance which turns into a thickened, yellowish, and opaque nail with considerable onycholysis. Unfortunately, there are still practitioners and surgeons that avulse ingrown nails. This is almost invariably followed by a recurrence. Nevertheless, some patients had to go through this inadequate and torturing procedure six times [36]. In our experience, nail avulsion for treatment of ingrown nail is not only useless, but it is almost always also harmful. Even for the treatment of infected granulation tissue, nail avulsion is not indicated.

A central strip of nail, 4 to 5 mm wide, may be removed without any incision into the soft tissue of the nail folds or nail bed [38]. This takes the outward pressure of the nail plate away and—according to the authors—allows the nail to grow out without piercing into the lateral grooves. It permits normal activities after about 3 days [38]. However, this should be accomplished with gauze or cotton packing in order to free the nail spicule from the nail groove.

#### 5.2.2. Wedge Excisions

**Figure 8:**
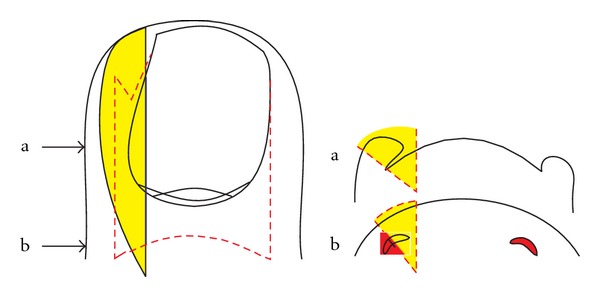
Schematic illustration how wedge excisions are most commonly performed; the wedge is very wide in the middle of the lateral nail fold, but the lateral matrix horn is not completely excised. (a) Transverse section at the level of the midnail bed, (b) transverse section at the level of the matrix horns.

**Figure 9:**
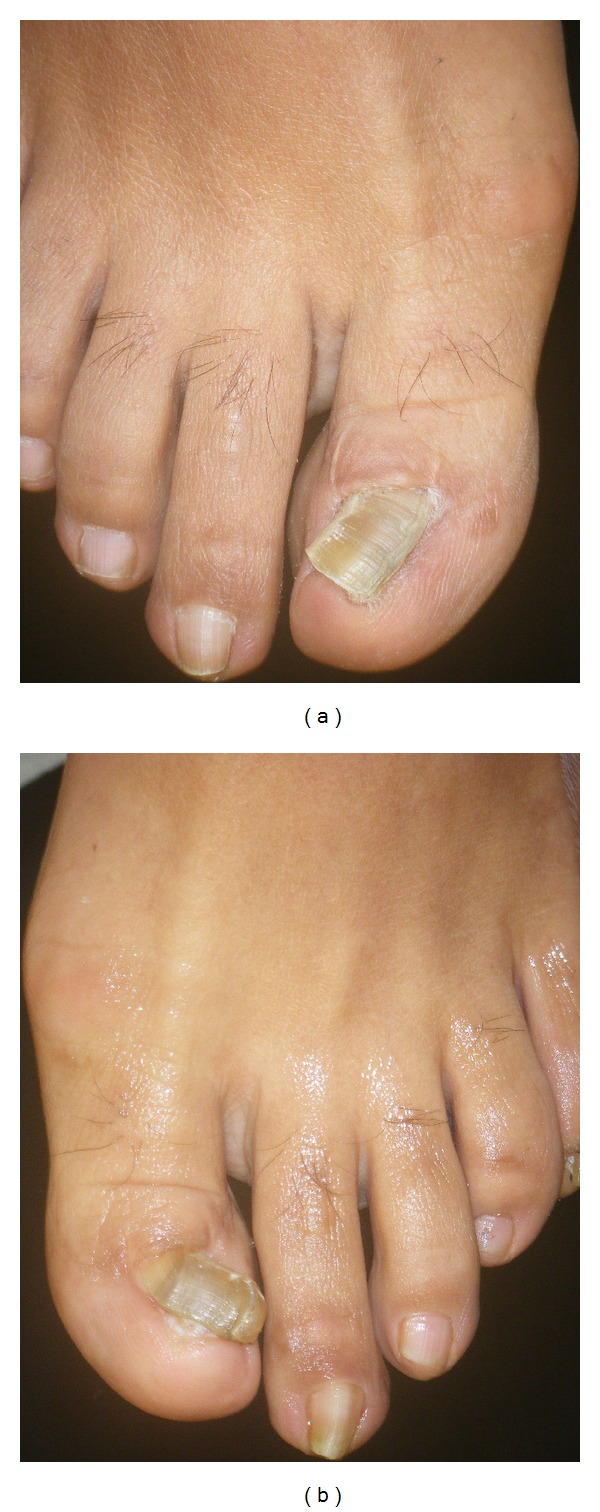
Toenails of a 38-year-old female patient 16 years after bilateral wedge excisions for ingrown nails showing onychogryphosis and malalignment. (a) Right foot, (b) Left foot.

Wedge excisions in their many minor variations do not consider the true shape of the matrix of the great toe, as is shown in most schematic illustrations of their authors [27, 39]. Most authors do not draw the correct shape of the matrix horns (Figure 8). Wedge excisions have a very high morbidity rate as healing of the wound takes 3 to 6 weeks in many patients. It is also mutilating as the lateral nail folds are removed and the nail is no more ensheathed by them. Often, the nail becomes dystrophic, particularly when the operation was carried out together with a nail avulsion. The nail will grow markedly narrow, distorted, onycholytic, thickened, discolored, and deviated (Figure 9). We therefore deem wedge excisions, whether they are carried out as Baudens', Emmert's, Kocher's, Watson-Cheynes', or McWilliams' operation, as being obsolete as they have a very high recurrence rate, a poor aesthetic and functional outcome, and an important morbidity.

**Figure 10:**
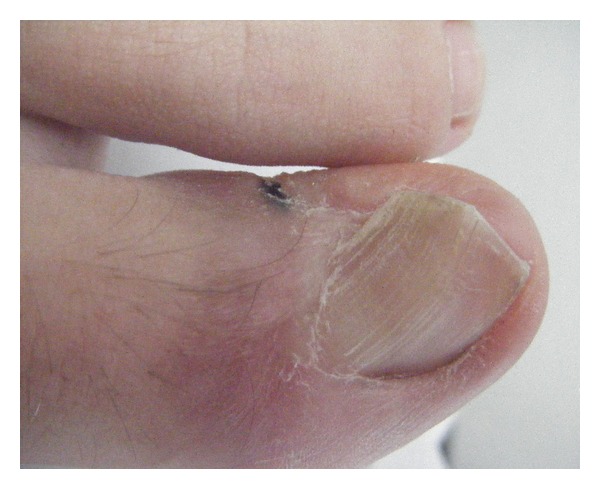
16-year-old boy 4 years after a wedge excision, which had been complicated by infection and necrosis of the lateral nail fold. There is considerable malalignment to the side of the necrosis.

Complications are frequent. There appears to be a risk of postoperative infection (Figure 10), and many surgeons give peri- or postoperative antibiotics. Even fungal septicaemia has been observed postoperatively [40]. A subtotal toe necrosis in a 10-year-old boy after Kocher's wedge excision was recently reported [41]. However, most toe necroses after ingrown nail surgery were due to a neglected tourniquet [42, 43].

Other authors described a “simple technique,” which involves wedge excision of the ingrowing nail, and bipolar diathermy of the nail bed [44]. It is not clear whether the authors really mean the nail bed or rather the matrix, which is responsible for the nail plate formation. They had to reoperate 9.9% because of recurrences, which is an unacceptably high rate.

#### 5.2.3. Reduction and Removal of the Lateral Nail Fold

**Figure 11:**
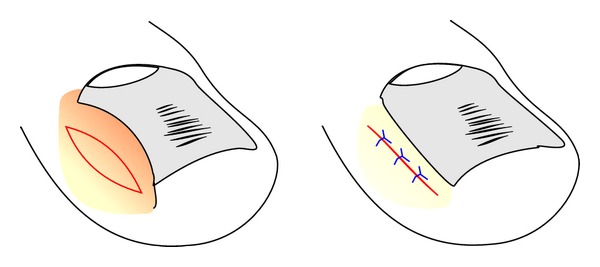
Schematic illustration of the reduction of a hypertrophic lateral nail fold by a fusiform excision.

As a consequence of the foreign body irritation by the ingrown nail, the lateral nail fold often becomes swollen, overlaps the lateral aspect of the nail plate, and develops granulation tissue. Over a long period, the nail fold becomes fibrotic and has no tendency to return to a normal size. Excision of a fusiform piece of skin from the lateral aspect of the distal phalanx and suture pulls the exuberant nail fold laterally and away from the nail (Figure 11) [45]. This has been slightly modified in that the ellipse has been turned into a crescent [46].

The Vandenbos technique takes out a big chunk of the soft tissue of the lateral nail fold down to the bone. After cauterization for haemostasis, the defect of approximately 1.5 by 3 cm is left for second intention healing. Neither the nail plate nor the matrix or nailbed are touched [27, 47]. The cosmetic results are very good; however, healing takes several weeks [48].

Noël's procedure is similar. The first incision is carried out from the middle of the distal lateral nail wall through the lateral nail groove up to one centimeter into the proximal nail fold. From there a second incision runs laterally to remove an elliptic wedge of soft tissue. The incisions are performed down to the lower third of the toe, to remove a large piece of soft tissue, but with preservation of some skin of the lateral aspect of the nail to permit direct closure with interrupted 4/0 stitches [49].

DuVries recommended to widely excise the lateral nail wall and subcutaneous fat and to suture the skin of the lateral aspect of the distal phalanx directly to the nail bed so that the nail lies on top of the skin and cannot dig into the hypertrophic nail fold because there is no sulcus left [50]. Ney's technique also is a generous excision of soft tissue [51].

Another radical soft tissue removal is Perez Rosa's super U [52]. In some respects, it is similar to Vandenbos' technique; however, it does not only remove the lateral nail walls, but also the soft tissue distal to the free nail margin resulting in a large U-shaped wound. In contrast to the aforementioned technique, the super U does not reach into the lateral aspect of the proximal nail fold. Haemostasis is achieved by a locked suture. Healing is by second intention and may take up to ten weeks. Improvement is excellent.

Howard proposed to remove a crescent of soft tissue from the tip of the toe parallel to the hyponychium [53]. A fishmouth-like incision is performed from one side of the tip to the other and another incision starting and ending at the same points like the first is made to yield a half-moon-shaped piece of tissue, which is excised down to the bone. By suturing the resulting wound, the hyponychium is pulled down abolishing the false distal nail wall and also pulling down the junction of the lateral nail groove with the distal nail groove, which is the most frequent site of ingrowing. This technique was redescribed about 80 years later [54] and appears to have been widely practiced in France.

A modification is the so-called lateral foldplasty [55]. A rectangular flap is formed from the most distal part of the lateral nail fold, and a triangular piece of skin is excised from the lateral part of the hyponychium. Skin is excised in addition below the flaps so as to pull down the junction of the distal groove with the hyponychium. This is in fact a modification of a hemilateral Howard operation.

#### 5.2.4. Excision of the Nail Bed

Quénu advocated a radical nail bed and matrix ablation [33]. This became later known as Zadik's procedure [34]. A comparative study showed 60.5% recurrences with Zadik's procedure [56]. In our opinion, this is an inadequate and far too radical method and in no case indicated.

#### 5.2.5. Amputation of the Tip of the Toe

The terminal Syme operation is in fact an amputation of the tip of the toe [36]. It involves resection of the nail bed and matrix, amputation of the distal half of the terminal phalangeal bone, and defect closure with a flap formed by the ridged skin of the tip of the toe. It results in a shortened, bulbous toe. As even this method is not free from recurrences, it is a mutilating and obsolete technique.

#### 5.2.6. Surgical Segmental Matrix Excision

**Figure 12:**
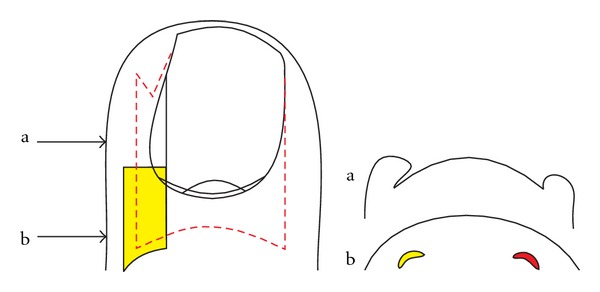
Schematic illustration of the selective lateral matrix horn resection.

Selective excision of the lateral matrix horn is a much less-invasive approach and respects the aetiopathogenesis of ingrown nails. It leads to a narrowing of the nail with a very high cure rate in ingrown nails. A nail elevator is inserted under the ingrown lateral strip of the nail to free it from the nail bed and then from the overlying proximal nail fold. The plate is cut straight back to the cuticle and under the nail fold to the proximal end of the matrix. An oblique incision is made at the junction of the proximal and distal nail folds, and the folds are reflected allowing the deep part of the lateral matrix to be seen. When the nail strip is taken out, the nail edge very often shows a sharp spike resulting from the improper nail cutting of the patient. The matrix horn with about 2 mm of the adjacent nail bed is meticulously dissected from the bone (Figure 12).

**Figure 13:**
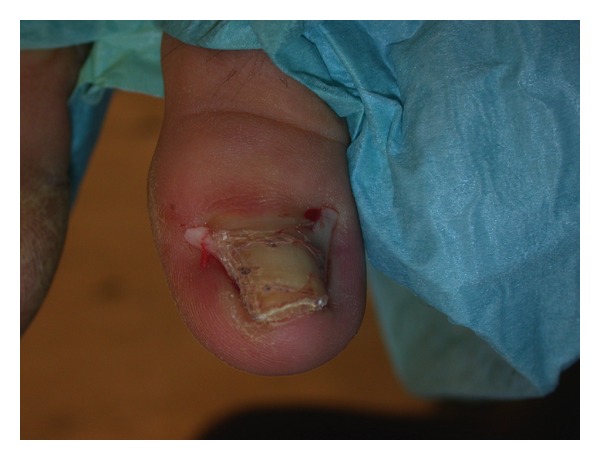
Proximal lateral and medial nail portions corresponding to the lateral matrix horns. The lateral nail strips have been separated from the nail bed, and the most proximal-lateral corners of the nail are elevated to show its true shape. As they are markedly curved downwards, the matrix horns are expected to reach deep plantarly and proximally.

The little wound is left open, but the nail walls are brought together either by simple stitches or suture strips (steristrips). We insert small tapered antibiotic tablets into the wound cavity that also contain lidocaine (Leukase Kegel) both for local antibiotic treatment, to reduce postoperative pain and above all to keep the space open to allow the wound secretion to escape. A padded dressing with an antibiotic ointment finishes the intervention. The patient is asked to elevate the foot for 24 to 48 hours. Healing is fast, usually in less than 10 days. The surgical matrix horn resection has a critical point. The most proximal corner of the matrix is usually very deep (Figure 13), and dissection may be difficult. Insertion of an injection needle [1, 57] and staining of the matrix horn with methylene blue [58] or gentian violet may aid in the dissection. Healing is usually faster than with phenol matricectomy though in one study it took longer [59].

**Electrocautery**

Instead of surgical dissection of the matrix horn, it may be cauterized electrosurgically or with a radiosurgery device [60]. Again, it has to be secured that no matrix horn remnants remain. The potential disadvantage is that classical electrocautery delivers a lot of heat that may eventually lead to a thermal periostitis with long-term postoperative pain.

**Laser**

A great number of publications deal with laser treatment of ingrown toenails. Almost invariably, the carbon dioxide laser was used to ablate the matrix horn [61–63]. The authors stress that the use of the CO_2_ laser is recommended because of markedly reduced pain, minimal disability, and satisfactory long-term results as well as shortened operation time due to minimal bleeding [64–66]. This was, however, contradicted by other authors who found a recurrence rate of 48% for partial and 50% for total matricectomy [67, 68]. Some authors also vaporize the lateral groove and granulation tissue [69]. The recurrence rate after resection of the nail segment and its nail bed alone was 37.5% whereas it dropped to 6.2% after additional lateral nail fold vaporization [70]. The erbium-YAG laser was also used for a modified wedge excision [71]. Other authors used the CO_2_ laser for haemostasis after surgical matrix horn resection [72].

#### 5.2.7. Segmental Matrix Horn Cauterization

**Phenol**

**Figure 14:**
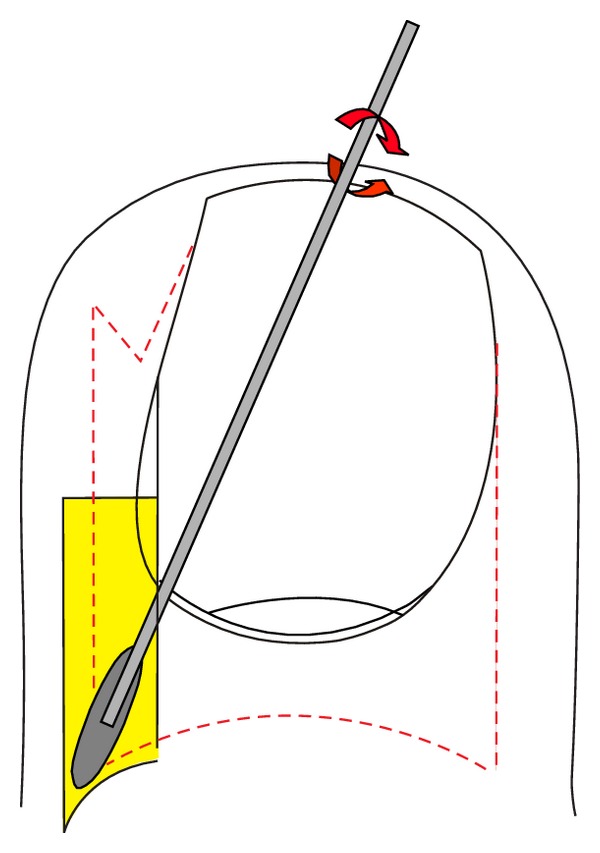
Schematic illustration of lateral matrix horn phenolisation. The ingrown strip of nail plate is avulsed, and a cotton tip applicator dipped into liquefied phenol is vigorously rubbed into the matrix horn under the proximal nail fold for 2 to 3 minutes.

**Figure 15:**
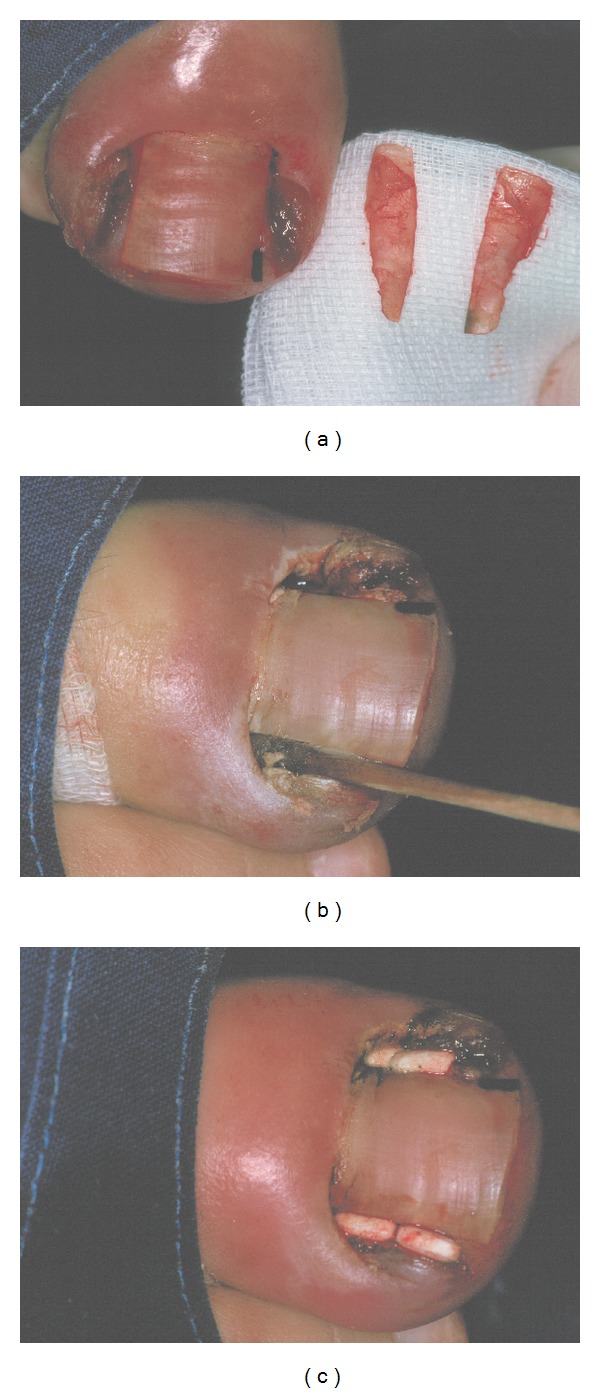
Phenolisation of the lateral matrix horn. (a) The lateral nail strips are avulsed and shown. (b) Phenol is rubbed into the lateral matrix horn. (c) At the end of surgery, small antibiotic tablets are put into the wound cavity.

Selective lateral matrix horn cauterization with liquefied phenol is now probably the most commonly used method. It is technically extremely simple, time-honouring, and safe with a recurrence rate between <1 and 2%. Liquefied phenol is made using 100 g of crystalline phenol, which is gently warmed in a water bath to about 45° when the crystals start melting. Under stirring 9.1 mL of distilled water is added dropwise. When the solution cools down to room temperature, the water-in-phenol solution remains liquid—hence it is called liquefied phenol—with a consistency approximately like glycerol. Phenol has three positive properties for the treatment of ingrown nails; it is a chemical cauterant thanks to its protein coagulating power, it is a potent disinfectant, and it has local anaesthetic activity. This reduces bleeding, makes postoperative infection very rare, and diminishes postoperative pain. Under local anaesthesia, either a proximal ring block or a distal wing block, the ingrown side of the nail plate is separated from the nail bed and the overlying proximal nail fold. The nail plate is cut straight forward till under the proximal nail fold and avulsed. This almost always shows a spike at the distal lateral end of the nail strip. A tourniquet is applied, any blood is dried, and a wisp of cotton is dipped into the liquefied phenol. It is then vigorously rubbed into the lateral matrix horn for about 2-3 minutes (Figures 14 and 15). Any granulation tissue may be gently touched with the phenol, but it will anyhow disappear spontaneously as soon as the offending lateral nail strip has been removed [1].

In a method so widely used, there are of course many small variations. Some authors prefer to swab the phenol treated area with alcohol in order to stop the phenol action [73–76]. This is dilution of the remaining phenol, but no neutralization [77].

Phenolization can be used in diabetics with the same complication rate as in nondiabetics [78]. It is not contraindicated in persons with impaired arterial blood supply.

In recent years, a debate was started about a possible infection risk and a delayed healing time after matrix horn phenolization. In our experience, infection after phenol cautery is extremely rare as we have never experienced infection even though we do not administer peri- or postoperative antibiotic prophylaxis or treatment, respectively. Phenolization causes a controlled necrosis of the matrix epithelium and subjacent connective tissue. This is the prerequisite for a successful therapy. It was shown that application times of 1, 2, and 3 minutes are effective with a recurrence rate of 12.9, 3.9, and 2.1%, respectively. Pain was identical in all three groups whereas oozing was longer in the 2- and 3-minute application times [79].

Phenolization can be safely used in children [80]. The success rate is about the same when the phenol cautery was performed by senior house officers [81].

Using lidocaine with epinephrine 1 : 100,000 was associated with significantly shorter healing times compared to plain lidocaine: 11.1 days versus 19.0 days, and less anaesthetic solution was required [82]. Also ferrichloride 20% after the phenol rub shortened the healing period [83].

Compared to cold steel surgery, phenolization of the matrix horn is much less painful, has a higher success and lower recurrence rate, and heals as fast or even faster than scalpel matrix excision [58]. However, in this study, phenol had a higher recurrence rate, which is in contradiction with more than 50 other reports [84].

Phenol is usually applied with a cotton-tipped applicator. In one study the authors proposed to use gauze instead and claimed that gauze use would minimize the risk of phenol burn of the surrounding skin [85].

Another issue under debate is the safety of phenol for the health personnel. This has been investigated, and the results were reassuring [86].

**Sodium Hydroxide**

Sodium hydroxide, either 10% or 20%, has been used since more than 20 years [87, 88]. The results are equally good [89] although some authors claim that postoperative drainage and healing times are shorter with sodium hydroxide [90–92], but others saw a longer healing time with NaOH than with Zadik's procedure [56]. Sodium hydroxide may also be used in diabetic subjects [93]. The necessary application times were studied, and 1 minute was found to be the optimal period concerning the success rate and the time to complete healing [87].

**Trichloroacetic Acid**

Recently 100% trichloroacetic acid was used to cauterize the lateral matrix horn. Success rate was 95%, and healing was complete within 2 weeks without prolonged drainage [94].

## 6. Controversies

As outlined above, there are many areas of debate. The first question is to whether treat conservatively or surgically. The noninvasive methods require consistent patient compliance and experience from the side of the treating physician. Among the surgical procedures, either narrowing of the nail or removal of the hypertrophic nail fold, or sometimes both, may be carried out. Judging from the literature [95] and own experience, selective matrix horn resection is the surgery of choice; which modality is used is of secondary importance provided it is radical enough. Recurrence rates vary in the literature reflecting different levels of experience.

## Figures and Tables

**Table 1:** Types of ingrowing nails.

Age of onset/growth direction	Common cause	Treatment
Neonatal	Free nail margin has not yet overgrown the tip of the toe	Conservative: massage
Infantile		
(1) Congenital malalignment of the big toenail	Malformation, probably genetic	Spontaneous healing in about 50%, if not by the age of 2 years: operation
(2) Hypertrophic lateral lip	Harmless malformation	Massage usually sufficient
Adolescent	Distal lateral ingrowing due to narrow nail bed	Conservative: packing, taping, gutter, acrylic nail; selective lateral matrix horn resection
Adult	Sharply bent lateral margin	Packing, gutter; surgical narrowing of the nail
Distal embedding	Big toenail too short	Taping of the distal nail wall, surgery
Retronychia	Chronic trauma with marked onycholysis leading to proximal ingrowing	(Proximal) nail avulsion
Pincer nail	Wide base of the distal phalangeal bone with large medial and smaller lateral osteophytes. Some drugs	Orthonyxia (braces)
		Narrowing of the nail, in severe cases with nail bed plasty
